# 白介素-17在肺癌发生及进展中的作用与机制研究进展

**DOI:** 10.3779/j.issn.1009-3419.2016.01.06

**Published:** 2016-01-20

**Authors:** 建东 梅, 伦旭 刘

**Affiliations:** 1 610041 成都，四川大学华西医院胸外科，中国西部肺癌早期诊断与综合治疗协同创新中心 Department of Toracic Surgery, West China Hospital, Sichuan University, Chengdu 610041, China; 2 Western China Collaborative Innovation Center for Early Diagnosis and Multidisciplinary Terapy of Lung Cancer, Chengdu 610041, China

**Keywords:** 白介素-17, 炎症, 肿瘤发生, 肿瘤进展, 肺肿瘤, Interleukin 17, Inflammation, Carcinogenesis, Cancer progression, Lung neoplasms

## Abstract

白介素-17（interleukin 17, IL-17）是一个重要的炎症因子，参与介导了机体的抗感染免疫及自身免疫性疾病相关的病理性炎症；此外，IL-17还与多种炎症相关的肿瘤有着密切联系。吸烟是导致肺癌的重要危险因素之一，而吸烟等因素所致的肺部慢性炎症反应伴有IL-17过表达，提示IL-17可能与肺癌的发生存在潜在联系；同时，IL-17还通过多种机制影响肺癌进展，本文对这一领域的相关研究进展进行了综述。

## Abstract (en)

Interleukin 17 (IL-17) is an important pro-inflammatory cytokine. It plays a critical role in mediating pathogen defense reactions, and the pathological inflammation of autoimmune diseases. IL-17 is also involved in various inflammation-related carcinogenesis. Cigarete smoking is one of the most important risk factors of lung cancer. Chronic inflammation caused by smoking and other factors is accompanied with overexpression of IL-17 within the airway, which reveals a potential relationship between IL-17 and lung carcinogenesis. Furthermore, IL-17 also plays a role in lung cancer progression via di?erent mechanisms. In this paper, we summarized the results of current studies on IL-17 and lung carcinogenesis, as well as lung cancer progression.

## 1 白介素(interleukin, IL)-17概述

IL-17是由Rouvier等^[1]^从小鼠淋巴样细胞cDNA文库中筛选发现，最初被命名为细胞毒性T淋巴细胞抗原8 (cytotoxic T lymphocyte antigen 8, CTLA-8)。Yao等^[2, 3]^证实CTLA-8是来源于CD4^+^T细胞的细胞因子，将其命名为IL-17。此后，多种与IL-17具有同源性的细胞因子陆续被发现，为加以区分，将IL-17又称为IL-17A^[4]^，IL-17家族其他成员包括IL-17B、IL-17C、IL-17D、IL-17E(亦称为IL-25)、IL-17F^[5-9]^，其中以IL-17F在结构和功能上与IL-17A最为接近，同源性高达55%^[4]^，但IL-17A的作用明显强于IL-17F^[10]^，其与受体的亲和力也高于IL-17F，IL-17F则可负调节IL-17A的表达^[11]^。IL-17家族的受体(IL-17 receptor, IL-17R)共发现IL-17RA、IL-17RB、IL-17RC、IL-17RD、IL-17RE等5个成员，其中，IL-17RA可分别与IL-17RB、IL-17RC、IL-17RD组成受体异二聚体^[12]^，而IL-17RA与IL-17RC组成的异二聚体是IL-17A及IL-17F的共同受体，两种受体缺一不可^[12, 13]^。

体内IL-17A的来源具有多样性。Park及Harrington等^[14, 15]^分别发现了CD4^+^T细胞中有别于T1及T2的特殊辅助T细胞亚群，即T17细胞，IL-17A及IL-17F为其标志性产物，这是对T细胞认识的一个重要突破。除Th17细胞外，CD8^+^T细胞、γδT细胞、自然杀伤(natural killer, NK)细胞等多种免疫细胞，甚至一些上皮细胞也可分泌IL-17A^[10, 16]^。

IL-17A作为IL-17家族最重要的炎症因子，目前研究最为深入，其参与了机体抗感染免疫、自身免疫性疾病相关的病理性炎症反应、肿瘤发生及进展等过程，但其与肿瘤的关系存在一定争议。一方面，IL-17A可通过促进血管内皮生长因子(vascular endothelial growth factor, VEGF)、转化生长因子β(transforming growth factor-β, TGF-β)等的表达促进肿瘤进展，另一方面，IL-17A也可通过激活细胞毒性T细胞(cytotoxic T lymphocyte, CTL)、NK细胞、中性粒细胞等发挥抗肿瘤作用^[17]^。对肿瘤临床标本的观察也发现IL-17A在不同肿瘤中作用的差别，例如肝癌及肺癌患者中，IL-17A高表达预示预后不良，而食管癌则与之相反^[18]^。肺癌是我国最为常见的恶性肿瘤，也是肿瘤致死的首要原因^[19]^，IL-17A作为一个炎症因子，可能参与了肺癌发生、进展的全过程，本文对目前的相关研究结果进行综述。鉴于文献中通常将IL-17A简称为IL-17，本文亦沿用了这一习惯名称。

## 2 IL-17促进肺癌发生及进展的临床证据

吸烟是肺癌及慢性阻塞性肺疾病(chronic obstructive pulmonary disease, COPD)最为重要的危险因素，且半数以上肺癌患者合并不同程度的COPD^[20]^。在肺功能正常的吸烟者及COPD患者肺组织中，IL-17阳性细胞数及IL-17表达水平均显著高于非吸烟者^[21, 22]^。此外，吸烟还与*IL-17*基因多态性相关，与IL-17F则无显著相关性，吸烟者携带至少一个拷贝*IL-17*基因G-152A位点等位基因时，患肺癌的风险增加2.06倍^[23]^。*IL-17*基因多态性还可上调IL-17的表达，这也增强了肺癌易感性^[24-26]^。肺癌患者肿瘤组织及外周血中IL-17也显著增高^[27-32]^，且IL-17高表达还与患者不良预后相关^[27-31]^，甚至肺癌患者呼出气体冷凝物中IL-17也有增高，其浓度与肿瘤大小正相关^[33]^，而外周血中IL-17浓度则与TNM分期正相关^[34]^。对于晚期肺癌患者，其恶性胸水中IL-17浓度也高于良性胸腔积液^[35, 36]^，发生脑转移时，患者外周血及脑脊液中IL-17浓度均显著增高^[37]^。与IL-17增高相伴的还有VEGF表达升高^[27, 28, 38]^，以及促淋巴管生成的VEGF-C表达上调^[31]^，且肺癌组织中IL-17的表达也与新生血管及淋巴管密度正相关^[27, 31, 39]^。对肺癌患者的观察提示IL-17可促进肺癌发生及进展，而其促进肺癌进展的可能机制之一是促进肿瘤脉管生成。

作为体内IL-17重要来源的Th17细胞，其在肺癌进展中的作用与IL-17可能不完全一致。肺癌患者恶性胸水及外周血中T17细胞数量显著增高^[37, 40-47]^，且胸水中T17细胞可能还参与了胸膜腔炎性微环境的维持^[40]^；但与IL-17高表达预示患者不良预后相反，胸水中Th17细胞数量与患者预后正相关^[48]^。Th17细胞可保护机体免受病原菌攻击，而调节型T细胞(regulatory T cell, Treg)则抑制机体对自身抗原的免疫反应，避免自身免疫的发生，二者在体内保持动态平衡^[49, 50]^。肺癌患者外周血中，Th17与Treg细胞比例均高于正常人，但二者在数量上呈负相关^[42]^；同时，恶性胸水及外周血中Treg与Th17细胞的比值高于健康人^[43-45]^，且比值与分期正相关^[45]^，与患者预后负相关^[43]^，即Treg细胞数量相对较多而Th17细胞数量相对较少与不良预后有关，尽管这些结果是在较小样本人群中的观察，依然可以提示Treg与Th17的失衡可能参与了肺癌的免疫逃逸及进展。基于对上述研究结果的综合分析，可以看出IL-17与Th17细胞在肺癌进展中的作用存在一定差异，但这一现象尚需在同一患者群体中进行较为系统的观察，以进一步明确二者在肺癌进展中作用的异同。

## 3 IL-17促进肺癌发生的机制

与吸烟人群相同，暴露于香烟烟雾中的小鼠肺部炎症反应也伴有分泌IL-17细胞的增多及IL-17表达水平的升高，给予抗IL-17抗体则可减轻香烟暴露小鼠肺部炎症反应^[51]^。肺部局限性*K-ras*突变小鼠易发生原发性肺癌，其肿瘤组织中也存在大量Th17及Treg细胞聚集，以非特异性流感嗜血杆菌裂解物(lysate of nontypeable Haemophilus inﬂuenza, NTHi)诱导该小鼠发生类似COPD的炎症反应，可导致肺组织中较多Th17细胞浸润^[52]^；然而，对于肺部特异性*K-ras*突变的*IL-17*基因敲除小鼠，无论是否给予NTHi刺激，其肺癌的发生均显著减少，肿瘤细胞增殖及新生血管也较IL-17野生型小鼠减少，伴有肺组织中促炎症分子IL-6、CCL2、Arg1、CSF3、基质金属蛋白酶7(matrix metalloproteinase-7, MMP7)、MMP12及MMP13等的分泌减少；另一方面，IL-17还通过诱导肺部CXCL2及G-CSF的表达募集CD11b^+^Gr1^+^髓系抑制细胞(myeloidderived suppressor cells, MDSCs)，MDSCs可促进肿瘤血管生成，并抑制CD8^+^T细胞及NK细胞的增殖与活化、诱导Treg细胞抑制机体免疫反应，从而促进肺癌发生与进展^[52]^。此外，Xu等^[53]^通过经口咽吸入搭载IL-17 cDNA的腺病毒，增强*K-ras*突变小鼠肺部IL-17的表达，在吸入病毒1周后，肺部IL-17的表达较对照小鼠高150倍，该组小鼠肺癌的发生也显著增多，且IL-17高表达还伴有MMP9表达的上调及肿瘤细胞侵袭能力的增强。上述研究结果从正反两方面证实了IL-17具有促进肺癌发生的作用，IL-17促进肺部免疫抑制微环境的形成，并增强肿瘤血管生成及侵袭性是其促进肺癌发生的重要机制。

## 4 IL-17在肺癌进展中的作用及机制

IL-17与肺癌进展及肿瘤耐药也存在密切关系，其可能参与了肿瘤进展相关的多重机制，例如血管生成、侵袭转移、免疫逃逸等；但也有部分研究显示IL-17在某些情况下还可能具有抑制肿瘤进展的作用，分别总结如下。

### 4.1 IL-17促进肺癌血管生成

对人肺癌组织标本的观察显示IL-17表达与肿瘤微血管及淋巴管密度正相关，提示其促进肿瘤血管生成的作用，体内外研究也证实了IL-17的这一作用。一方面，IL-17可直接促进肿瘤微血管生成。肿瘤缺氧微环境的代谢产物乳酸可上调IL-17的表达^[54]^，IL-17作用于肺癌细胞，可诱导其高表达多种血管生成相关的CXC趋化因子，包括CXCL1、CXCL5、CXCL8等，且IL-17刺激肺癌细胞后的条件培养基还可趋化血管内皮细胞^[55]^，转染了IL-17 cDNA的肺癌细胞株在免疫缺陷小鼠体内成瘤后的生长也更为迅速，高表达IL-17的肿瘤组织中血管密度更高，通过抗体中和上述趋化因子的受体CXCR-2，可抑制IL-17诱导的肿瘤血管生成并延缓其生长^[55]^，结合这些结果，可以推测IL-17通过募集血管内皮细胞，并通过上述多种趋化因子与CXCR2结合，共同促进血管生成。此外，IL-17也可促进部分肺癌细胞株高表达VEGF^[38, 56]^，但单纯阻断VEGF并不影响IL-17所诱导的肿瘤生长^[55]^。另一方面，IL -17还与肿瘤抗血管生成治疗耐药有关。Chung等^[57]^分别观察了肺癌、结肠癌、淋巴瘤等的皮下移植瘤，以及肺癌原位移植瘤模型，发现肿瘤组织中浸润的Th17细胞分泌IL-17，通过NF-κB通路上调粒细胞集落刺激因子(granulocyte colony-stimulating factor, G-CSF)的表达，募集MDSCs至肿瘤微环境，导致抗VEGF治疗耐药，而对IL-17受体敲除小鼠移植瘤模型给予抗VEGF治疗，或对普通小鼠模型联合抗VEGF与抗IL-17治疗，均可抑制肿瘤对抗血管生成治疗的耐受。

### 4.2 IL-17促进肺癌侵袭转移

IL-17可通过多种途径促进肺癌转移灶的形成，对于*IL-17*基因敲除小鼠，经尾静脉注射肿瘤细胞后的肺部转移灶明显少于野生型小鼠^[34, 58]^。肺部炎症环境及*IL-17*的表达还可促进转移灶的生长，同样经尾静脉注射小鼠Lewis肺癌细胞建立肺转移瘤模型，给予香烟烟雾及NTHi刺激增强肺部炎症反应，可促进肿瘤细胞增殖，但IL-17基因敲除小鼠则不受此影响，且肿瘤进展较IL-17野生型小鼠缓慢^[59]^。在IL-17促进肿瘤转移的机制方面，IL-1可能扮演着关键角色，Carmi等^[58]^通过经尾静脉注射Lewis细胞建立肺转移瘤模型，证实肿瘤微环境通过IL-1募集γδT细胞，分泌IL-17并促进转移灶形成。对乳腺癌肺转移模型的观察还发现IL-17通过G-CSF活化中性粒细胞，共同促进肺及淋巴结转移灶的形成^[60]^。此外，转录因子T-bet具有抑制IL -17表达并抑制肿瘤进展的作用，IL-17的表达与*T-bet*负相关，而与Treg细胞转化因子Foxp3的表达正相关，对T-bet基因敲除小鼠给予抗IL-17抗体干预，亦可延缓经尾静脉注射Lewis细胞所形成的肺部转移灶进展^[61]^。

上皮间质转化(epithelial-mesenchymal transition, E MT)是肿瘤转移的另一重要机制。I L -17可诱导肺癌细胞株A549发生EMT相关标志物表达的变化，如诱导波形蛋白(vimentin)高表达，同时抑制E粘连蛋白(E-cadherin)的表达，增强肿瘤细胞的侵袭性，其分子机制是IL-17通过激活NF-κB通路，诱导转录抑制物ZEB1的表达，从而促进EMT的发生^[62]^。此外，体外研究^[63]^发现IL -17还可能通过促淋巴管生成促进肿瘤转移，其机制是通过细胞外调节蛋白1/2(extracellular signal-regulated protein kinase 1/2, ERK 1/2)通路，促进Lewis细胞及A549细胞分泌促进淋巴管生成的重要因子VEGF-C，并趋化淋巴上皮细胞，从而促进淋巴管生成。此外，IL-17还可促进肺癌细胞分泌MMP2、MMP9等^[53, 64]^，也为肺癌侵袭转移创造了条件。

### 4.3 IL-17诱导肺癌免疫抑制微环境

MDSCs是粒细胞、巨噬细胞、树突状细胞等的前体细胞，肿瘤微环境通过IL-17募集较多MDSCs^[52, 57]^，抑制机体抗肿瘤免疫^[57]^。同时，IL-17还参与促进肺癌形成以M2型肿瘤相关巨噬细胞(tumor-associated macrophages, TAMs)为主的肺癌肿瘤微环境。一方面，肺癌微环境通过高表达IL -17募集TAMs^[32]^，另一方面，IL -17还上调肺癌细胞环氧化酶2 (cyclooxygenase-2, COX2)的表达，进而增加前列腺素E2 (prostaglandin E2, PGE2)的合成，促进M2型TAMs的分化^[65]^，而M2型TAMs具有免疫抑制的作用，可通过促进肿瘤血管生成、侵袭转移等多种途径促进肿瘤进展。

### 4.4 IL-17抑制肺癌进展的证据

尽管上述较多研究支持IL-17促进肺癌发生及进展，目前也有一些证据提示IL-17在某些情况下还可能抑制肿瘤进展。Lin等^[66]^采用Lewis细胞建立小鼠恶性胸腔积液模型，发现*IL-17*基因敲除小鼠的肿瘤进展更快，生存期也显著缩短，IL-17的缺失抑制了Stat1通路的激活并促进了T1细胞的活化；IFNγ的缺失则抑制Stat3通路的激活，并促进Th17细胞的活化，抑制恶性胸水形成，延长小鼠生存期。人恶性胸水可通过CCL20及CCL22等募集Th17细胞，由IL-1β、IL-6、IL-23等诱导其分化成熟，发挥抑制肿瘤进展的作用^[48]^。同时，一些抗肿瘤治疗也需要IL-17及Th17细胞的参与才能发挥作用。大剂量脂多糖可抑制肺转移性黑色素瘤，该过程也伴有较多T1及T17细胞的活化^[67]^，而对黑色素瘤肺转移模型的观察发现敲除*IL-17*基因可加速肿瘤进展，Th17细胞则可激活肿瘤特异的CD8^+^T细胞，从而抑制肿瘤进展^[68]^。此外，Marshall等^[69]^联用PI3K通路抑制剂与Toll样受体激动剂治疗小鼠Lewis肺癌，发现上述制剂发挥作用需IL-17及干扰素γ(interferon γ, IFNγ)的参与，若IL-17缺失，则上述制剂无法抑制肿瘤生长。IL-17与Th17细胞在肿瘤进展中的作用并不完全相同，具有抗肿瘤作用的Th17细胞可能属于肿瘤抗原特异性Th17细胞^[70]^。此外，肺移植瘤中的IL-17主要来源于γδT细胞^[58]^，机体内共生菌的刺激可维持该群细胞的活化，维持其对肿瘤的杀伤作用^[71]^。由此可见，IL-17及Th17细胞亦可能直接或间接参与了对部分移植瘤的抑制作用。

## 5 小结

慢性炎症与肺癌的发生密切相关，通过对肺癌高危人群、肺癌患者的观察及动物实验，均证实重要炎症因子IL-17可促进肺癌发生；此外，IL-17还通过促进肿瘤血管生成、促进其侵袭转移及诱导肿瘤免疫抑制微环境等促进肺癌进展，但对肺癌患者的观察却显示出Th17细胞抑制肿瘤进展的潜力。同时，采用肺癌移植瘤模型所进行的研究则显示IL-17促进与抑制肺癌进展的双重作用，为进一步解决这其中的争议，排除移植瘤中免疫等因素的干扰，在未来的研究中尽量采用原发性肺癌模型，或可为明确IL-17在肺癌进展中的作用提供更具说服力的证据。基于现有研究结果，尝试通过干预IL-17或Th17细胞治疗肺癌尚缺乏充分证据。
